# Recognition of Ionic Liquids as High-Voltage Electrolytes for Supercapacitors

**DOI:** 10.3389/fchem.2020.00261

**Published:** 2020-05-05

**Authors:** Shanshan Pan, Meng Yao, Jiahe Zhang, Bosen Li, Chunxian Xing, Xianli Song, Peipei Su, Haitao Zhang

**Affiliations:** ^1^Beijing Key Laboratory of Ionic Liquids Clean Process, CAS Key Laboratory of Green Process and Engineering, Institute of Process Engineering, Chinese Academy of Sciences, Beijing, China; ^2^School of Chemical Engineering, University of Chinese Academy of Science, Beijing, China; ^3^Hebei Institute of Process Innovation Co. Ltd, Langfang, China

**Keywords:** ionic liquids, high-voltage electrolytes, liquid electrolytes, ionogel electrolytes, supercapacitors, mechanisms

## Abstract

The electrochemical stability of electrolytes is essential to the working potential of supercapacitors. Ionic liquids (ILs) are being considered as safe alternatives to current organic electrolytes and attracting extensive interests owing to their inflammability, widened potential windows, and superior ionic conductivity. Novel supercapacitors with IL electrolytes exhibit attractive energy density and can be utilized in various energy storage systems. Most previous studies focused on electrochemical performances, while rare attentions were devoted to energy storage process details or mechanisms. This review comprehensively summarizes the latest progress on formulated IL electrolytes for different types of supercapacitors, with an emphasis on the intrinsic understanding of the related energy storage mechanisms. Subsequently, comparisons of various IL-based liquid-state electrolytes as well as the state-of-the-art advancements in optimizing ILs electrolytes are introduced. The authors attempt to reveal the inherent correlation between the usage of IL electrolytes and the properties of supercapacitors via referenced works. Some emerging applications of ionogel electrolytes based on conventional polymers and poly(IL)s for flexible supercapacitors are also presented, including the existing problems. In addition, challenges and future perspectives of research in this field are highlighted.

## Introduction

Electrochemical energy storage systems have attracted extensive interests in recent years due to their widespread applications in smart electronics, electric vehicles, as well as hybrid load-leveling systems for intermittent green sources. Among various energy storage devices, lithium-ion batteries (LIBs) and supercapacitors (SCs) are the two most extensively used energy storage systems (Ding et al., 2018; Li M. et al., 2018). Nowadays, commercial LIBs with low power density and potential safety issues cannot meet the growing energy needs. Fortunately, SCs with rapid power output and long cycle life can be used independently or supplement batteries in many fields such as electric buses, light rail, wearable electronics, and energy storage systems for intermittent renewable energy sources, triggering growing tremendous interests (Conway and Pell, 2003; Simon and Gogotsi, 2008; Shao et al., 2018). Thus, it is highly desirable to understand the mechanisms of supercapacitors and develop advanced supercapacitors.

SCs can store and release charges rapidly since the charging and discharging processes are limited by surface processes rather than ion diffusion. Generally, SCs can be classified into three categories: electric double-layer capacitors (EDLCs), pseudocapacitors, and hybrid supercapacitors. Energy density, as one of the main parameters, could evaluate the properties of supercapacitors. However, low energy density hampered the widespread commercialization of supercapacitors. The energy density (*E*) of supercapacitors is determined by voltage window (*V*) and capacitance (*C*) of the device according to Equation 1 (Simon and Gogotsi, 2008):

(1) $$\begin{array}{l} E=\frac{1}{2}CV^{2} \end{array}$$

On the one hand, the energy density of supercapacitors can be elevated by developing electrode materials with high specific capacity; on the other hand, a significant increase in energy density can be achieved by widening the potential window of the device as the energy density is proportional to *V*^2^. The potential window of the device is mainly limited by the electrochemical stability of the used electrolytes. One means of raising the voltage is to employ organic electrolytes with higher decomposition voltage instead of aqueous electrolytes. Unfortunately, organic electrolytes suffered from inflammability and toxicity, resulting in serious safety issues. Therefore, developing safe and high-voltage electrolytes can promote the performances of SCs.

Compared to aqueous and organic electrolytes, ionic liquids (ILs) composed of a discrete anion and cation have many merits, including widened electrochemical stability window (~4.5 V), superior ionic conductivity, negligible volatility, and low flammability (Armand et al., 2009; Van Aken et al., 2015; Watanabe et al., 2017). Therefore, ILs are promising alternatives to traditional electrolytes of supercapacitors and thus have attracted widespread attentions. Till now, various IL-based electrolytes including pure ILs, eutectic ILs, and ILs/organic solvent mixtures have been extensively investigated in supercapacitors. Most of IL electrolytes previously studied were non-amphiphilic ILs. In the published literatures so far, non-amphiphilic ILs based on imidazolium and pyrrolidinium were extensively researched due to the relatively lower viscosity and reasonable ionic conductivity (Balducci et al., 2004; Galinski et al., 2006). In general, imidazolium salts possess high ionic conductivity, while pyrrolidinium salts exhibit wide electrochemical stability windows. More recently, Mao et al. discovered surface-active IL electrolytes for EDLCs, exhibiting unusual interfacial ion distributions owing to significant van der Waals interactions, which enhanced the performance of EDLCs (Mao et al., 2019). Furthermore, IL electrolytes allow safe operation at higher temperatures due to their outstanding thermostability, and one of the first demonstrations of this was that of Mastragostino's group (Largeot et al., 2011). Although tremendous efforts have been devoted to IL electrolytes for supercapacitors and much progress has been made, IL electrolytes still have some drawbacks such as high viscosity and limited ionic conductivity at low temperatures and poor contact with electrodes, which greatly limited their applications in supercapacitors (Balducci et al., 2005). Forming eutectic IL mixtures is considered as an effective route to improve the low temperature performance of supercapacitors, which violates the traditional concept that IL electrolytes can only be used above room temperature. The extended temperature range of −50 to 100 °C for a supercapacitor was achieved by employing an eutectic IL mixture (1:1 Pip_13_FSI:Pyr_14_FSI) (Lin et al., 2011). In addition, with the increased consumption of flexible electronic devices, IL-based solid or gel electrolytes are the most promising electrolyte candidates for solid supercapacitors (Lu et al., 2014). Compared to typical solid electrolytes, gel electrolytes exhibit better flexibility, higher ionic conductivity, as well as acceptable compatibility with electrode, being suitable as electrolytes for flexible supercapacitors. More importantly, ILs are not only widely employed in energy storage devices but also as model materials/electrolytes to solve fundamental problems caused by increased capacitance and ion transfer in carbon nanopores. Besides these actual applications, IL media are direct systems for basic research to understand the properties of the electrodes/electrolytes interface in supercapacitors because there is no solvent. Unfortunately, the cost issue is still a problem in considering commercial applications, which is due to the purification cost, not synthesis.

**Figure 1:**
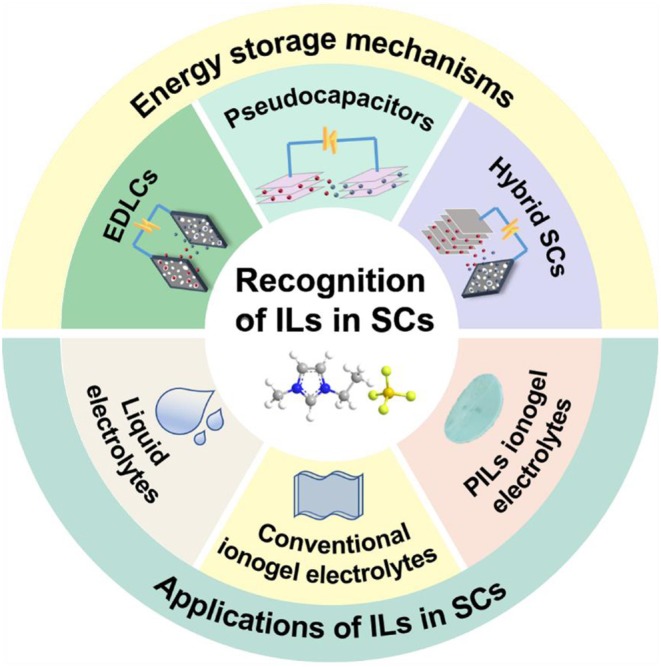
Energy storage mechanisms and applications of ionic liquids (ILs) in supercapacitors (SCs).

Although ILs have price and purity issues, their significant advantages as electrolytes are undeniable. Exploiting advanced IL electrolytes matching with nanostructured carbon is certainly a promising direction for constructing the next generation of high-energy density supercapacitors with broad temperature range. In addition, the design of novel electrolytes based on ILs is of vital importance to the standardization of electrochemical tests and methods, which can evaluate the stabilities of electrolytes employed in supercapacitors. So far, electrolytes based on ILs for supercapacitors have been widely investigated with the purpose of widening the potential window of devices, and some related reviews have been made (Yin et al., 2019). However, there are limited reviews concerning the intrinsic mechanisms of IL-based supercapacitors. The latest research progress also needs to be concluded, which will provide the guidance for future studies of supercapacitors with IL electrolytes. In this review, storage mechanisms of different kinds of supercapacitors will be explained in order to understand the behaviors of IL electrolytes in different types of supercapacitors. We emphatically present the recent advances in liquid-state IL electrolytes and IL-based solid electrolytes for supercapacitors (Figure 1). We also focus on the common improvement strategies of IL electrolytes and the latest developments. In addition, some remaining challenges for future supercapacitors are enumerated.

## Energy Storage Mechanisms

**Figure 2:**
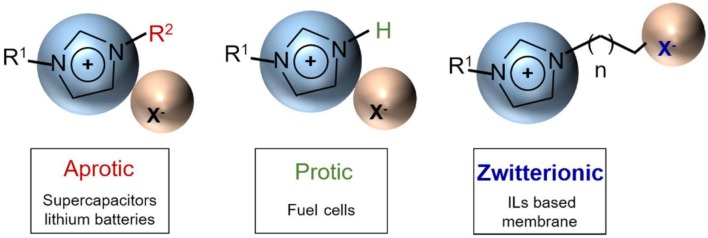
Three categories of ionic liquids (ILs) (aprotic, protic, and zwitterionic) and their corresponding applications. Adapted from Armand et al. (2009), Nature Publishing Group.

ILs play a crucial role in the field of energy storage devices such as lithium ion batteries, supercapacitors, and fuel cells, usually as their electrolytes. Different energy storage devices have diverse basic principles and different requirements for IL electrolytes. ILs are generally divided into three categories: aprotic, protic, and zwitterionic, as shown in Figure 2. Among them, aprotic type ILs are suitable as high-voltage electrolytes for supercapacitors and lithium ion batteries, while protic ILs can be applied to fuel cells (Armand et al., 2009). Understanding the mechanisms of ILs in various supercapacitors including electric double-layer capacitors, pseudocapacitors, and hybrid supercapacitors is important for guiding the selection and engineering optimization of IL electrolytes.

### Electric Double-Layer Capacitors With ILs

**Figure 3:**
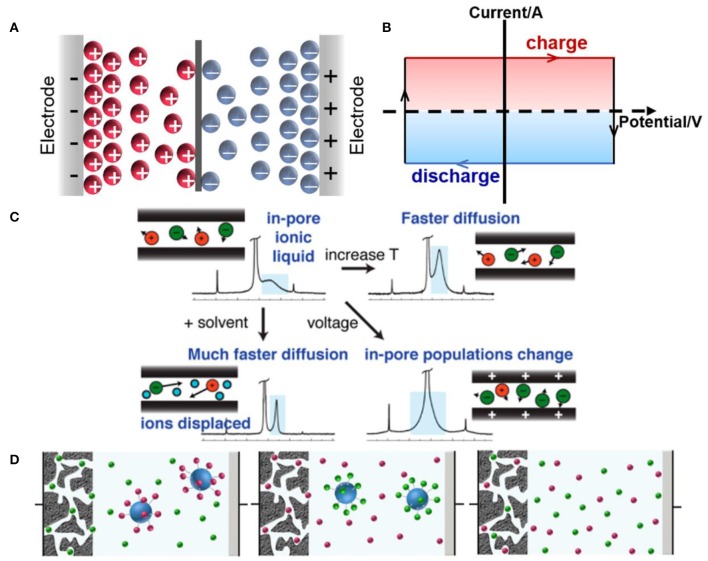
Schematics of **(A)** charge-storage mechanisms and **(B)** cyclic voltammogram curve of ideal EDLCs; **(C)** charging mechanism of in-pore ionic liquids (ILs) and the effects of temperature, solvent, and voltage on ions dynamic. Reproduced from: **(C)** Forse et al. (2015), American Chemical Society, and **(D)** Dou et al. (2017), Nature Publishing Group.

EDLCs are the simplest supercapacitors based on charge separation process, which stores energy by the charge arrangement in the Helmholtz bilayer. Figure 3A shows the typical double-layer charge storage mechanism. The cyclic voltammogram curve of ideal EDLCs should maintain a rectangular shape when the solution resistance is ignored, which shows a typical capacitive behavior, as illustrated in Figure 3B. The charge storage process in EDLCs only involves physical absorption and desorption on the surface of electrodes, without the occurrence of Faradaic reaction (Conway, 1991; Stoller and Ruoff, 2010), thus providing high power density. The stored energy of EDLCs is restricted by the specific surface areas of the electrode materials, and carbon-based materials with high specific surface areas are usually selected as the electrodes of EDLCs (Liu et al., 2010; Simon and Gogotsi, 2013). Capacitance is the physical characteristic of the electrodes/electrolytes coupling performance for EDLCs, which can be described as Equation 2 (Zhang and Zhao, 2009):

(2) $$\begin{array}{l} C_{dl}=\frac{Q}{V}=\frac{\varepsilon_{r}\varepsilon_{0}}{d}A \end{array}$$

where *C*_dl_ is the capacitance of one electrode of EDLCs, *Q* is the charge transferred at a applied voltage *V*, ε_r_ is the dielectric constant of the electrolyte, ε_o_ is the dielectric constant of vacuum, *d* is Debye length, which represents the effective charge separation distance, and *A* is the surface area of electrodes. When *C*_dl_ is constant for EDLCs, the following Equation 3 describing the response current *I* can be derived from the above Equation 2:

(3) $$\begin{array}{l} I=\frac{dQ}{dt}=C_{dl}\frac{dV}{dt} \end{array}$$

In Equation 3, *t* is the charge time. If the potential *V* changes linearly with time *t*, $\frac{dV}{dt}$ can be considered as a constant and denoted as γ. As a result, Equation 3 can be simplified to Equation 4:

(4) $$\begin{array}{l} I=C_{dl}\gamma \end{array}$$

Up to now, tetraethylammonium tetrafluoroborate (Et_4_NBF_4_) in organic carbonates is the most widely used electrolytes (Pandolfo and Hollenkamp, 2006). However, the electrochemical stability window of organic carbonates is lower than IL system, limiting the energy density of EDLCs. The energy density of EDLCs can be elevated to that of Ni/MH batteries (>100 W h/kg) by employing IL electrolytes with widened voltage window (Liu et al., 2010; Tamailarasan and Ramaprabhu, 2012), which is much higher than that of supercapacitors with organic electrolytes. However, the structure of electric double layer in IL media with no solvent is different from that in traditional electrolytes since the solvent molecules will distribute charged ions. Kornyshev proposed the classical model, Gouy–Chapman–Stern (GCS), was inaccurate for many of the basic assumptions are inadequate (Kornyshev, 2007). Especially, GCS model not only ignores the correlation between ions but also treats all ions as a point charge. It was okay for organic or aqueous system, but in ILs, the relationship between anions and cations was the center of physical properties of ILs. Thus, he put forward a new theory called mean-field theory accounting for the finite size of ions and the mutual interactions between ions (Bazant et al., 2012), which showed that different differential capacitance curves would appear in different electrolytes. Pean proposed a simulation methodology using a hybrid supercapacitor simulation cell to compute a single-electrode capacitance in pure IL electrolytes at 100°C (Pean et al., 2015). For the IL system, the strong correlation between ions of ILs is the main obstruction in standalone electrolytes consisting of ILs. Some groups diluted the IL electrolytes to maintain ions of ILs separated in order to address the above issue. Recently, the first insight of IL interface using various experimental techniques was reported. By atomic force microscopy (AFM), a strong delamination effect was observed at the interface of electrodes/electrolytes (Atkin and Warr, 2007; Hayes et al., 2010; Atkin et al., 2011). Later, this was confirmed by surface force apparatus (SFA) (Smith et al., 2013) or by high-energy X-ray reflectivity (Mezger et al., 2008, 2015).

However, the above studies focused on the interfacial properties between ILs and planar electrodes. Compared to planar electrodes, porous carbons are the most widely used due to their high accessible surface area. Burt et al. used a combined electrochemical/simulation method to elucidate the behaviors and mechanisms of pure ILs and its mixture with acetonitrile (ACN) in nanoporous carbons (Burt et al., 2016). The results indicated that higher concentrated ions of pure ILs did not result in significant increase in capacitance of nanoporous carbon-based EDLCs, which was attributed to the increased difficulty in separating ions of opposite charges of pure ILs. The mechanism of EDLCs changes from counterion adsorption in diluted ACN to ion exchange in pure ILs. In addition, the interface structure is affected by the structure of the electrode surface and IL electrolytes. Planar electrodes show a layered structure with ordered or disordered layers of ions, while electrodes with defects may break the layered structure. EDLCs with nanoporous carbons exhibit the best performance (Salanne, 2017). Remarkably, the introduction of IL electrolytes may not lead to the obvious increase in capacitance, but the structures of electrodes affect the capacitance. Till now, great efforts have been devoted to improving the energy density of EDLCs in consideration of the matching of pore size and IL size (Chmiola et al., 2006). From Jose's research, he showed that the size of bulk ILs mostly depends on the length of the alkyl chain of the ions (Lopes and Padua, 2006). Furthermore, Liu demonstrated that the influence of the alkyl chains of ILs on the electrochemical properties of graphene nanosheets was studied by electrochemical experiments (Liu et al., 2011). More recently, the performance of ILs in electrode models of different structures was investigated using molecular dynamics simulations by Pereira et al. (2019). For a planar electrode, ILs formed a layered structure that changed with the polarity of the electrode due to the asymmetry of ions. For nanoporous electrodes, a monolayer of ions inside smaller pores was observed, while a multilayered structure was observed in wider pores. They also proposed that higher energy density was achieved by employing electrodes with narrower pores at lower voltages. However, at high potentials, supercapacitors employing electrodes with wider pores exhibited higher energy density.

The obvious advantages of ILs are widened voltage window and high thermostability, ensuring high energy density and wide operating temperature range of EDLCs. A wide electrochemical window (up to 3.6 V) and good high-temperature durability of the 1-ethyl-3-methylimidazolium and *N, N*-dimethyl pyrrolidinium cation-based electrolytes were demonstrated by Zhu et al., exhibiting a good application prospect in supercapacitors (Zhu et al., 2007). Besides, some new ILs have been employed, such as azepanium used for supercapacitors (Pohlmann et al., 2015). However, compared to organic electrolytes, conventional ILs exhibit no significant increase in capacitance. In addition, the power performance of ILs cannot compete with organic electrolytes. Therefore, Forse et al. used nuclear magnetic resonance (NMR) spectroscopy to study ion dynamics and charge storage of ILs in porous carbons, thus explaining the power properties of ILs in EDLCs. They found that the mechanism of IL-based EDLCs involved both counterion adsorption and coion desorption. In addition, the effects of temperatures, voltages, and addition of organic solvent on the rate of ionic diffusion were investigated (Figure 3C). Elevated temperatures and the addition of solvent enhanced ionic mobility, and higher applied voltages changed the in-pore populations of ions. Faster diffusion meant more rapid power output (Forse et al., 2015). Thus, the power density could be improved by developing ILs with faster ionic diffusion. Recently, this group observed ion dynamics in supercapacitors during charging via *in situ* NMR measurements, indicating that in-pore ion populations, ion–ion interaction, and carbon porous structure would affect ion dynamics (Forse et al., 2017). These works may be favorable for the adjustment of the energy and power density of IL-based supercapacitors. In addition, Dou et al. monitored cations and anions independently using silica nanoparticle-grafted ILs, as shown in Figure 3D; silica nanoparticle-grafted ions cannot enter the pores of activated carbon, whereas free counterions can enter upon charging (Dou et al., 2017).

### Pseudocapacitors With ILs

The mechanism of pseudocapacitors is based on fast Faradaic redox reactions that occur at the surface or near-surface region of the electrode materials with electron transfer but without any bulk phase transformation during charging/discharging (Conway, 1995). The key difference between EDLCs and pseudocapacitors is that the former store energy by charge separation precisely like traditional physical capacitors, while the energy storage mechanism of the latter is exactly similar to that of battery systems. No chemical transformation occurred during charging and discharging process of pseudocapacitors, but due to the Faraday reaction, a reversible functionalized molecular layer was formed on the surface of the active materials. It is worth noting that the features of pseudocapacitors are completely different from the redox reactions involved in battery systems where bulk phase transformation occurs. The term pseudocapacitance is used to describe the capacitance, which comes from capacitive electrode materials undergoing fast electron-transfer Faradaic reactions. Thus, pseudocapacitance is not the real capacitance arising from double-layer charging but the pseudocapacitance generated by redox reactions without phase transformation.

**Figure 4:**
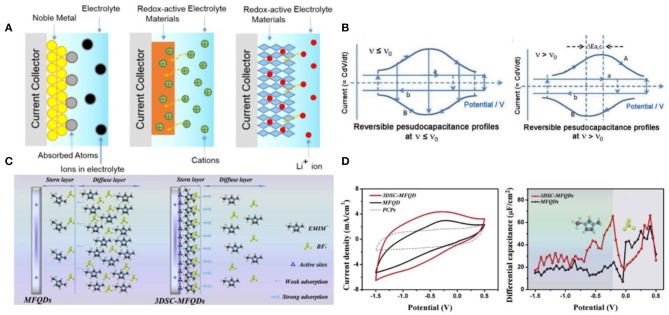
**(A)** Schematic diagrams of three different energy storage mechanisms of pseudocapacitors; **(B)** cyclic voltammetry (CV) profiles of reversible pseudocapacitors at different sweep rate *v*, where *v*_0_ is the critical sweep rate; **(C)** schematic diagrams of Stern/diffusion layer and **(D)** Cd–E curves of MFQDs and 3DSC-MFQDs. Reproduced from: **(A)** Augustyn et al. (2014), Royal Society of Chemistry; **(B)** Conway (1991), Electrochemical Society; and **(C,D)** Yang et al. (2020), Elsevier.

Pseudocapacitance was divided into three categories: (1) underpotential deposition, (2) surface redox system, and (3) intercalation system, as illustrated in Figure 4A (Conway, 1991; Conway and Pell, 2003; Augustyn et al., 2014). From the cyclic voltammetry (CV) curves of reversible pseudocapacitors in Figure 4B (Conway, 1991), the presence of critical sweep rate (*v*_0_) is one typical feature of pseudocapacitors because the presence of effective equivalent series resistance causes polarization effect. In addition, the charge transfer resistance limits the kinetic of pseudocapacitors, and the behavior of pseudocapacitors will deviated from ideal capacitive behavior. The kinetic reaction of pseudocapacitors would change from reversible to irreversible with the increase in sweep rate.

As we all know, ions (e.g., H^+^ or Pd^2+^) in solution can be adsorbed on the surface of the noble metal (e.g., Pt or Au) to form a monolayer under their equilibrium potentials for cation reduction, and the process was called underpotential deposition, which can be described by Equation 5 (Sudha and Sangaranarayanan, 2002):

(5) $$\begin{array}{l} M+xC^{z+}+xz^{+}↔C.M \end{array}$$

where *M* is the noble metal, *C*^*z*+^ is the deposited ions, *x* is the number of deposited ions, and *z* is the charges of the deposited ions. The capacitance produced by this process is usually limited due to the narrow potential ranges (0.3–0.6 V), which results in low energy density.

The surface redox system undergoes a Faraday reaction with electron transfer, such as transition metal oxides (e.g., RuO_2_, MnO_2_, conductive polymers) (Subramanian et al., 2004; Jiang et al., 2012a,b; Xia et al., 2012). The first pseudocapacitive material was RuO_2_, delivering a specific capacitance of over 800 F g^−1^. However, RuO_2_ suffers from the disadvantage of high cost, which still cannot be settled (Mayrand-Provencher et al., 2010). MnO_2_ is the promising low-cost electroactive material to replace RuO_2_ due to its versatility in the electrochemical behaviors. Meanwhile, ILs exhibited well performance in pseudocapacitors based on MnO_2_. Unfortunately, the specific capacitance is noticeably low (~100 F g^−1^) (Li and Wei, 2012; Li et al., 2012). Some groups used X-ray photoelectron spectroscopy, X-ray absorption spectroscopy, and electrochemical quartz crystal microbalance to reveal that large cations of IL electrolytes only adsorb on surface without permeation into the lattice structure of MnO_2_ (Benedetti et al., 2008; Chang et al., 2009; Lee et al., 2010). Compared with conventional electrolytes, ILs cannot widely dispersed within the pores of solid materials. In addition, the electronic conductivity of these metal oxides is rather low, and the wettability of electrode materials in ILs determines the charge transfer resistance. In addition, some groups found that many pseudocapacitors with IL electrolytes suffered from a huge iR drop (Brandt and Balducci, 2014; Rai et al., 2014; Navathe et al., 2015). One effective strategy is to use gold-modified electrodes, which can markedly promote the pseudocapacitive behaviors of active materials in ILs. Another way is to design the nanostructured active materials geometrically in order to match the interacting ions of electrolytes, which can achieve an energy density of 163 W h kg^−1^ of MnO_2_-based pseudocapacitors (Maiti et al., 2015). More recently, Yang et al. developed a 3D space-confined MnFe_2_O_4_ exhibiting superior interfacial properties and pseudocapacitive storage (Yang et al., 2020). They studied the mechanism of the Stern/diffusion layer of IL ions near electrode surface and concluded that the characteristic adsorption between this electrode and EMIM^+^ led to huge amounts of EMIM^+^ absorbed densely in the Stern layer, which enhanced capacitance, as shown in Figures 4C,D.

Intercalation pseudocapacitance is another Faraday process that occurs without crystalline phase transition and when the intercalation of quasi-two-dimensional electroactive substances occur, as illustrated in Equation 6:

(6) $$\begin{array}{l} BA_{y}+xLi^{+}+xe^{-}↔Li_{x}BA_{y} \end{array}$$

where *BA*_*y*_ is the intercalation electrode material (e.g., Nb_2_O_5_), and *x* is the transferred electrons number. The difference between this intercalation process for pseudocapacitors and batteries is whether the electrode materials undergo a crystal phase transition during electrons transfer. An embedded system in a tantalum capacitor includes inserting Li^+^ into the bulk of materials such as MoS_2_ (Yang et al., 2015; Yoo et al., 2016).

These three mechanisms of pseudocapacitors are based on different Faraday processes and appear in diverse materials; however, they provide similar thermodynamic characteristics such as the logarithmic relationship shown in Equation 7.

(7) $$\begin{array}{l} E=E^{0}+\frac{RT}{nF}\;\ln\frac{X}{\left(1-X\right)} \end{array}$$

where *E* is the electrode potential (V), *R* is the ideal gas constant (8.314 J mol^−1^ K^−1^), *T* is the temperature (K), *n* is the number of electrons, *F* is Faraday's constant (96,485 C mol^−1^), and *X* is the occupancy fraction of the surface or lattice layer.

Although the electrochemical responses of pseudocapacitive and double layer charging are similar, their mechanisms are totally different. Shen proposed a possible energy storage mechanism of γ-FeOOH electrode in IL electrolytes (EMIM-NTF_2_) (Shen et al., 2016). If the cations of ILs intercalate into the lattice, the size and the geometry of the organic cations should match the pores and channels of the lattice. Compared with the simple inorganic cations, the organic cations of ILs are larger, while IL cations have an asymmetrical charge distribution.

### Hybrid Supercapacitors With ILs

Hybrid supercapacitors can maximize the operating voltage window of supercapacitors via employing one battery-type anode and one capacitive-type cathode and thus have attracted extensive efforts (Li B. et al., 2018). Until now, lithium and sodium ion capacitors are the mostly investigated hybrid supercapacitors. Furthermore, using IL electrolytes can also widen the potential window of hybrid supercapacitors (dos Santos et al., 2019). Unlike IL electrolytes in EDLCs or pseudocapacitors, lithium or sodium salt is usually added to IL electrolytes in hybrid supercapacitors.

#### Lithium Ion Capacitors

Lithium ion capacitors (LICs), as a typical representative of hybrid supercapacitors, employ capacitive-type positives with large surface area and battery-type negatives with high capacity and thereby include both the intercalation/deintercalation and rapid adsorption/desorption charge–discharge mechanisms. During the charge and discharge process, ions were adsorbed/desorbed on the surface of the capacitive-type electrodes, while Li^+^ were intercalated/deintercalated in the bulk of battery-type electrodes. Electrolytes are of importance to the inherent storage mechanism and overall performance of LICs. IL electrolytes with high ionic conductivity and non-volatility in a wide temperature range as well as widened electrochemical windows can promote the performance of LICs. Therefore, tremendous studies were focused on ILs, which can be employed as the electrolytes of LICs.

**Figure 5:**
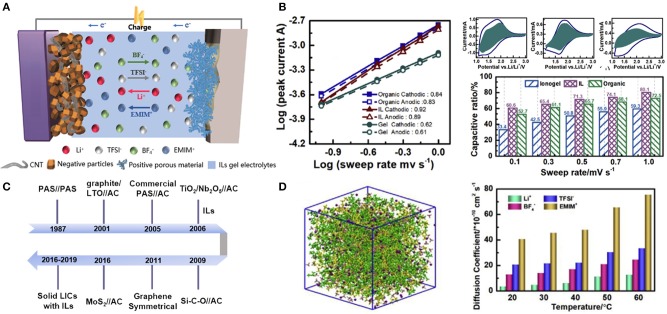
**(A)** Schematic illustration of ionic liquid (IL)-based lithium ion capacitors (LICs) structure; **(B)** specific current peaks of the three electrolytes (organic, IL, and IL gel) and their corresponding capacitive contribution with different sweep rates; **(C)** the evolution history of LICs; **(D)** simulated microstructures and dependence of the diffusion coefficients of different ions on temperature in the formulated IL electrolyte. Reproduced from: **(B,D)** Zhang J. H. et al. (2019), Elsevier.

Most of published works focused on electrode materials of LICs systems but rarely on electrolytes especially IL electrolytes and their transport mechanisms. The ions of ILs distinctly differ from solvated anions of conventional electrolytes, which would affect both the structure of double layer and the nature of ILs. The schematic of IL-based LICs configuration and the development history are illustrated in Figures 5A,C. Unfortunately, high viscosity of IL electrolytes may lead to poor rate capability compared to that of common organic electrolytes. Norihisa Handa et al. reported an IL electrolyte of 1-ethyl-3-methyl imidazolium bis(fluorosulfonyl)imide (EMIMFSI) for EDLCs, exhibiting superior rate capability regardless of whether the electrode materials contained a binder or not. In addition, employing EMIMFSI as electrolyte can inhibit self-discharge behavior. In contrast, the charge/discharge process of LICs includes Li^+^ intercalation/deintercalation mechanism, which is different from typical EDLCs. Therefore, additional lithium salts are needed to provide Li^+^. Previous work reported an LiFSI/EMIMFSI electrolyte for LIBs, showing well cycle durability and rapid charge–discharge performance. These results may be favorable for the design of IL-based electrolytes for LICs. More recently, an IL electrolyte system of LiFSI/EMIMFSI for LICs was proposed by Hirota et al. (2018). The LiFSI/EMIMFSI electrolyte is more stable than LiPF_6_-based solvent electrolyte at both high and low temperatures.

To comprehensively understand the effect of electrolytes on energy storage mechanism, the respective contributions of the capacitive- and Faradaic-type elements in three different electrolytes (organic, the formulated IL, and IL gel) were quantitatively calculated by Zhang J. H. et al. (2019), as shown in Figure 5B. The group found that the formulated IL (EMIMBF_4_) system gave the highest *b* value, indicating that the capacitive contribution was the most significant in the IL system. Furthermore, a simulation of the molecular dynamics was conducted to explain why the formulated IL enhanced capacitive contributions. Figure 5D illustrated that the diffusion coefficient of Li^+^ was the lowest among all ions in the IL electrolyte, which was due to the form of Li^+^ clusters. In contrast, EMIM^+^ possessed a relatively high diffusion coefficient and thus enhancing capacitive contributions. Through this work, we can get a good understanding of the internal storage mechanism of LICs with IL electrolytes.

#### Sodium Ion Capacitors

In consideration of the limited resources of lithium, sodium ion batteries (SIBs) are considered one of the most promising alternative energy storage systems due to the adequate resources and low cost of sodium (Shen et al., 2018). Inspired by the research progress of SIBs and LICs, immense research interests have been focused on sodium ion capacitors (SICs). SICs feature the similar mechanism and kinetics to LICs, except that the transport ions (Na^+^ or Li^+^) and anions in the electrolytes are different. The ionic radius of Na^+^ is larger than Li^+^, which may result in sluggish ion diffusion dynamics and large volume expansion of electrode materials. Electrode modification including structural optimization and carbon modification have been proposed to address the above disadvantages. Stettner et al. investigated the possibility of using IL-based electrolytes for SICs (Stettner et al., 2018). Mixtures of 1-butyl-1-methylpyrrolidinium bis(trifluoromethanesulfonyl)imide (Pyr_14_TFSI), 1-butylpyrrolidinium bis(trifluoromethanesulfonyl)imide (PyrH_4_TFSI), and bis(2-methoxyethyl)ether diglyme solvent were fabricated and applied to sodium-based devices, showing attractive prospects for future sodium-based systems. In addition, NaTFSI has been used as conducting salt to ensure that the electrolyte is suitable for sodium-based systems. Presser's group conducted proof-of-concept study of hybrid supercapacitors lithium titanate (LTO)/AC with ILs/LiTFSI or NaTFSI electrolytes (Fleischmann et al., 2019).

## IL-Based Liquid Electrolytes in Supercapacitors

**Figure 6:**
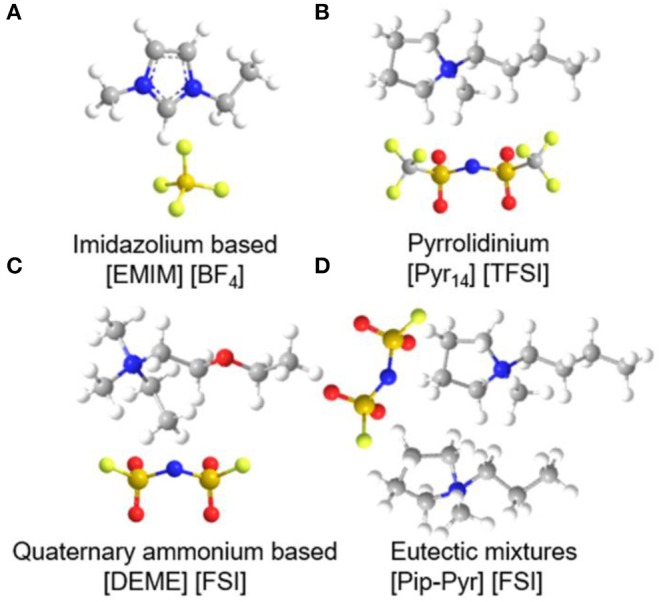
The molecular structure of four kinds of common ionic liquids (ILs): **(A)** imidazolium-, **(B)** pyrrolidinium-, **(C)** quaternary ammonium-based, and **(D)** eutectic mixtures IL electrolytes.

The properties of electrolytes play a vital role in determining the overall performance of supercapacitors. Room-temperature ILs (RTILs), as liquid electrolytes for energy storage devices, have attracted tremendous efforts due to their advantageous ionic conductivity, electrochemical stability, and non-flammability. ILs based on imidazolium, pyrrolidinium, as well as aliphatic quaternary ammonium salts were the extensively used in supercapacitors, which will be discussed in detail below. Recently, eutectic IL mixtures are considered as the most promising electrolytes for supercapacitors at extreme temperatures compared to imidazolium, pyrrolidinium, and quaternary ammonium-based ILs (Lin et al., 2011). The molecular structure of common IL electrolytes are shown in Figure 6. The following section will focus on these four kinds of ILs and their applications in supercapacitors, as well as some effective strategies of improving the performance of IL electrolytes.

### Categorization and Comparison of Various Liquid IL Electrolytes

#### Imidazolium-Based Electrolytes

Immense research efforts have been aimed at imidazolium-based ILs owing to their low viscosity, high conductivity, and other special physicochemical properties, showing potential applications in supercapacitors. Most of the imidazolium-based ILs investigated previously are non-amphiphilic, while recently surface-active IL electrolytes have been applied to supercapacitors, which exhibits broad implications for supercapacitors.

##### Non-amphiphilic IL Electrolytes

**Figure 7:**
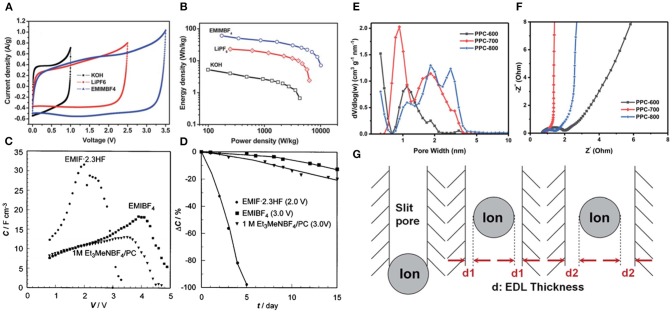
**(A)** Cyclic voltammetry (CV) profiles and **(B)** Ragone plots of the prepared electrode in different electrolytes; **(C)** voltage dependence of capacitances and **(D)** cycle test of electric double-layer capacitors (EDLCs) with various electrolytes at 70 °C; **(E)** pore size distribution and **(F)** Nyquist plots of carbon samples carbonized at different temperatures; **(G)** schematic diagrams of ion matching pores with different diameters. Reproduced from: **(A,B)** Lei et al. (2013), Royal Society of Chemistry; **(C,D)** Ue et al. (2003), Electrochemical Society; **(E–G)** Zhang L. et al. (2019), Royal Society of Cemistry.

Among various imidazolium-based RTILs, 1-ethyl-3-methylimidazolium (EMIM^+^)- and 1-butyl-3-methylimidazolium (BMIM^+^)-based electrolytes have been extensively investigated. Lei et al. used KOH, LiPF_6_, and 1-butyl-3-butyl imidazole tetrafluoroborat (BMIMBF_4_) as electrolytes for the reduced graphene oxide and CMK-5 composites electrode (RGO-CMK-5) based supercapacitors (Lei et al., 2013). Based on CV tests (Figure 7A), EMIMBF_4_ featured the widest electrochemical window, followed by LiPF_6_, and KOH electrolytes possessed the narrowest. As a consequence, the supercapacitor with EMIMBF_4_ electrolytes exhibited higher specific energy density than the supercapacitors using LiPF_6_ and KOH electrolytes (Figure 7B). However, the high viscosity of ILs especially at low temperatures is one of the key obstacles that hamper their industrial applications. 1-Ethyl-3-methylimidazole fluoride (EMIF·2.3HF) electrolyte with relatively low viscosity and superior low-temperature properties for supercapacitors was reported by Ue et al. (2003) In this work, the double-layer capacitor employing EMIF·2.3HF manifested higher capacitance than EMIMBF_4_ and the others even at low temperatures but showed lower energy density due to its lower decomposition voltage (Figure 7C). At 70 °C, the cycle performance and thermal stability performance of EMIF·2.3HF electrolytes were not ideal (Figure 7D), coupled with HF toxicity. In order to further improve the electrical conductivity of imidazolium-based IL electrolytes and reduce their viscosity, while maintaining a wide electrochemical window, some convenient strategies including mixing imidazolium-based ILs with hydrophobic proton organic solvents have been proposed. Dagousset et al. fabricated three binary mixtures of ILs (EMIMTFSI, Pyr_13_FSI, and Pyr_14_TFSI) and organic solvents γ-butyrolactone (GBL) and investigated their electrochemical characteristics in supercapacitors within a wide temperature range (−50 °C; +100 °C) (Dagousset et al., 2017). EMIMTFSI/GBL electrolytes presented the most excellent cycling performance (only 6.6 % loss of capacitance after 500 h) at −50 °C, which may be attributed to the remarkably low temperature stability of these mixtures. On the contrary, different results were obtained at high temperature of 100 °C. Pyr_14_TFSI electrolytes showed better stability at higher temperature. Although the mixtures of ILs/organic solvents could reduce the viscosity and enhance durability in severe environments, this is not a universal strategy, and the wide electrochemical windows of ILs would be sacrificed. More recently, Zhang et al. developed the porous carbons with high specific surface area and suitable micropore diameter to match IL ions (Zhang L. et al., 2019). They emphasized the importance of ion matching porous carbons in providing an ion highway for rapid ion transport to form more effective electric double layers, which resulted in superior performance (Figures 7E–G).

##### Surface-Active IL Electrolytes

**Figure 8:**
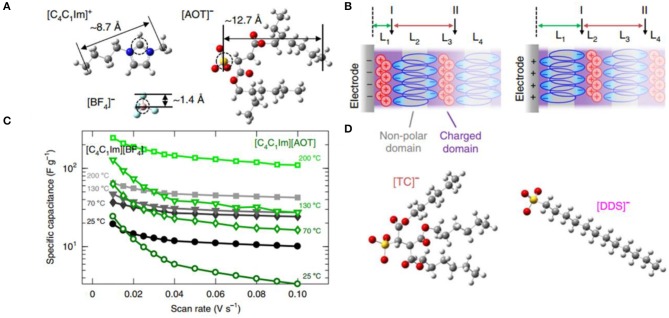
**(A)** Molecular structures of [C_4_C_1_Im]^+^, [BF_4_]^−^, and [AOT]^−^; **(B)** the specific capacitance vs. the scan rate for [C_4_C1Im][BF_4_] and [C_4_C_1_Im][AOT] at 25, 70, 130, and 200 °C; **(C)** schematics of hypothesized electric double-layer (EDL) structures of [C_4_C_1_Im][AOT] at negatively and positively charged interfaces with U = ±2 V; **(D)** molecular structures of [DDS]^−^ and [TC]^−^. Reproduced from: **(A–D)** Mao et al. (2019), Nature Publishing Group.

Surface-active ILs (SAILs) are an emerging IL class with surface active cations or anions, which can form self-assembled nanostructures at ILs/electrodes interfaces. More recently, Mao et al. first investigated the electrocapacitive characteristics and novel EDL structures of SAIL electrolytes (Mao et al., 2019). This work revealed that the self-assembly of SAILs could facilitate the energy storage of supercapacitors. The comparison of electrochemical properties and interfacial nanostructures between non-amphiphilic ILs (NAILs) (C_4_C_1_IMBF_4_) and SAILs (C_4_C_1_IMAOT) was performed by simulations and experiments (Figure 8). C_4_C_1_IMAOT exhibited larger capacitance than C_4_C_1_IMBF_4_ at higher temperatures, which may be due to their different EDL structures. NAILs at interface showed a typical layered structure of alternating charges, while SAILs exhibited an unusual structure with charged polar domains and non-polar domains of bilayer ions due to the self-assembly of the non-polar groups. For C_4_C_1_IMAOT, the polar head of [AOT]^−^ was attracted to the interface and the non-polar tails outwards, close to non-polar of the next [AOT]^−^ layer, preventing overscreening effects. Therefore, the presence of an [AOT]^−^ bilayer squeezed excess cations into the first ion layer near the interface to generate abundant free counterions concentration and form thinner EDLs and thus enhancing the capacitive performance. Furthermore, the group also investigated other SAILs with [DDS]^−^ and [TC]^−^, exhibiting similar EDL structures to that of C_4_C_1_IMAOT and thereby indicated that these phenomena are universal features of SAILs. Notably, SAILs showed larger capacitance under higher temperatures and wider voltage windows.

#### Pyrrolidinum-Based Electrolytes

In addition to imidazolium-based RTILs, pyrrolidinum-based RTILs belonging to cyclic quaternary ammonium salts were also widely applied to the electrolytes of supercapacitors. The asymmetry of substituting pyrrolidine cation resulted in low melting point and high conductivity. *N*-Butyl-*N*-methylpyrrolidine (trifluoromethyl sulfonyl) imide salt (Pyr_14_TFSI) has attracted a lot of attention for its excellent electrochemical and thermal stability at high temperature. An AC/Pyr_14_TFSI/pMeT hybrid supercapacitor with long cycle life and high voltage was constructed by A. Balducci et al. (2006). This supercapacitor delivered energy density of 24 W h kg^−1^ and power density of 14 kW kg^−1^ as maximum values at 10 mA cm^−2^ and 60 °C. M. Lazzari et al. studied the interfacial behaviors of Pyr_14_ TFSI and EMIMTFSI electrolytes on the surface of AC electrode, indicating that the capacitance of AC electrode was largely determined by the polarization of the cations of ILs when cathode was charged (Lazzari et al., 2007). The porosity and chemical properties of carbon materials play crucial roles in influencing the conductivity and polarization of ILs. Recently, Xu et al. synthesized novel anionic surfactant ILs Pyr_14_AOT, which was mixed with Pyr_13_TFSI to form electrolytes for supercapacitors (Xu et al., 2019). The highest specific capacitance was achieved for 10 wt% Pyr_14_AOT in Pyr_13_TFSI at 150 °C, which was attributed to the improved wettability by surfactant ILs. However, the larger non-polar of surfactant ILs resulted in reduced conductivity and poor performance at low temperatures.

#### Quaternary Ammonium-Based Electrolytes

In comparison with imidazolium-based ILs, the primary competitive advantage of quaternary ammonium-based ILs with short chain is their outstanding stability to large specific surface area of AC electrode. The electrochemical and physicochemical properties of *N, N*-diethyl-*N*-methyl-*N*-(2-methoxyethyl) ammonium tetrafluoroborate (DEME-BF_4_) electrolytes for EDLCs were investigated by T. Sato et al. (2004). DEME-BF_4_ electrolytes could facilitate the performance of electrochemical capacitors owing to their extremely widened potential window (6.0 V) and high ionic conductivity (4.8 mS cm^−1^ at 25 °C) in practice compared to EMIMBF_4_ electrolytes.

#### Eutectic IL Mixtures Electrolytes

**Figure 9:**
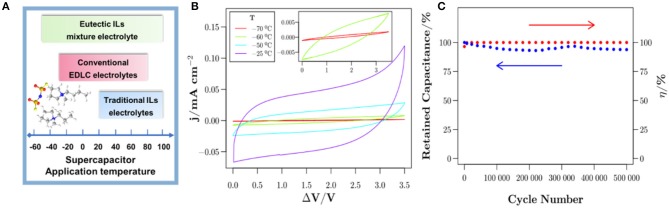
**(A)** Operating temperature of supercapacitors with different electrolytes system. **(B)** Cyclic voltammetry (CV) curves at low temperatures at a scan rate of 1 V s^−1^ and **(C)** cycling stability at a current density of 0.6 mA cm^−2^ of Ni-foam supercapacitor at room temperature. Reproduced from: **(A)** Lin et al. (2011), American Chemical Society; **(B,C)** Newell et al. (2018), Elsevier.

Eutectic IL mixtures are also promising candidate electrolytes for supercapacitors due to their lower viscosity and good stability. More importantly, eutectic ILs can broaden the range of temperatures for supercapacitors (Figure 9A). Recently, a novel eutectic IL mixture of EMIMTFSI and 1-propyl-3-methylpyrrolidinium bis(trifluoromethylsulfonyl)imide was fabricated by Rodrigo Newell et al. (2018). From Figure 9B, the conducted electrochemical measurements revealed that the eutectic IL mixtures electrolytes were suitable for supercapacitors with a cell voltage of 3.5 V at a wide temperature range from −70 to 80 °C. The Ni-foam supercapacitor assembled with such electrolytes exhibited superior cycling stability at a current density of 0.6 mA cm^−2^ and room temperature, as shown in Figure 9C. Mahanta et al. studied the eutectic electrolytes of 1-butyl-3-methyl imidazolium methanesulfonate ([BMIM][MeSO_3_])/N-methylacetamide (NMAc) (Mahanta et al., 2020). The results indicated that [BMIM][MeSO_3_]/NMAc showed higher thermal stability and lower viscosity compared to pure ILs but higher internal resistance.

### Improvements of IL Electrolytes

Although ILs have many advantages compared with conventional liquid electrolytes, their low degree of dissociation, high viscosity, as well as poor power performance need to be improved. Various ways such as adding organic solvents or carbon materials and introducing redox-active species to ILs have been proposed to optimize IL electrolytes. Wong et al. studied IL electrolytes with different amounts of acetonitrile (AN) in order to obtain optimum electrolytes (Wong et al., 2020). They concluded that 4 M EMIMBF_4_/AN achieved the highest capacitance, and pure ILs achieved the highest energy density. Nagao et al. proposed BaTiO_3_ additive, which could boost the capacitance of electrodes and enhanced the rate capability of EDLCs with EMIMBF_4_ electrolytes (Nagao et al., 2007). Compared with EDLCs without BaTiO_3_, EDLCs employing BaTiO_3_ powder/activated carbon composite as electrodes and the EMIMBF_4_ as electrolytes delivered larger capacitance and better rate performance. The positive effect of BaTiO_3_ was attributed to the fact that it could increase the dissociation degree of EMIMBF_4_. Then, the carrier concentration to form electric double layer would be increased, and the performance of EDLCs would be enhanced.

## IL-Based Solid Electrolytes in Supercapacitors

Solid supercapacitors show broad applications prospect in many fields such as safe energy storage devices and flexible electronics (Deng et al., 2019) because there are no issues of leakages and explosions. Obviously, the demand of non-liquid electrolytes for solid supercapacitors is growing. Gel electrolytes, consisting of a polymer skeleton as a host, an organic/aqueous solvent as a plasticizer, and a supporting electrolyte salt (Sekhon, 2003), are most widely used as non-liquid electrolytes that function both as separators and ion carriers in supercapacitors. Since Fuller et al. began to explore gel electrolytes based on ILs, much efforts have been devoted to the applications of IL gel electrolytes for various electrochemical systems, especially for quasi-solid lithium ion batteries and supercapacitors (Fuller et al., 1997). In the past years, polymer matrix doping ILs have been demonstrated to improve the thermal stability of gel electrolytes and expand the electrochemical window of the devices to 3.5–4.0 V (Fuller et al., 1998; Lyu et al., 2016).

Ionogel electrolytes are fabricated by trapping ILs into the polymer matrix. Supercapacitors utilizing ionogel electrolytes are regarded as quasi-solid supercapacitors due to the presence of ILs with some degree of fluidity into the polymer matrix. Generally, conventional neutral polymers and poly(IL)s (PILs) are widely used as the solid polymer matrix. Various methods including solution casting, electrostatic spinning, and *in situ* polymerization of various monomer in ILs medium have been used to obtain ionogel electrolytes.

### Conventional Polymer-Based Ionogel Electrolytes

The properties of ILs and polymer matrixes and the interaction between them play an important role in determining the ionic conductivity and electrochemical stable windows of ionogel electrolytes. Till now, various conventional polymers including poly(ethyl oxide) (PEO), poly(vinylidene fluoride) (PVDF), and poly(vinylidene fluoride-co-hexafluoropropylene) [P(VDF-HFP)] have been extensively investigated as polymeric host materials of ionogel electrolytes for energy storage devices. Most of the efforts were devoted to the ionogel electrolyte-based EDLCs and pseudocapacitors. There was also more and more attention paid to the investigation of emerging LICs employing ionogel electrolytes. Furthermore, based on ionogel electrolytes, flexible supercapacitors were developed.

**Figure 10:**
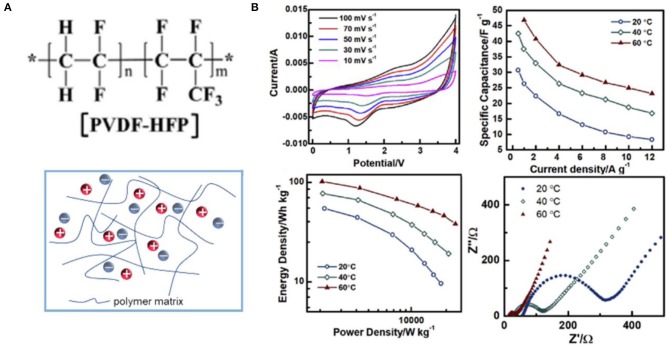
**(A)** Structural representation of poly(vinylidene fluoride-co-hexafluoropropylene) [P(VDF-HFP)] and P(VDF-HFP)-based ionogel electrolytes; **(B)** electrochemical performance of solid lithium ion capacitors (LICs) with P(VDF-HFP)–EMIMBF_4_-LiTFSI ionogel electrolytes. Reproduced from Zhang J. H. et al. (2019), Elsevier.

Among various polymers that can be used as the skeleton of ionogel electrolytes for supercapacitors, P(VDF-HFP) is the most popular polymer due to its low glass conversion temperature and strong mechanical strength (Ramesh and Ling, 2010). The chemical structures of P(VDF-HFP) and ionogel electrolytes based on P(VDF-HFP) are illustrated in Figure 10A. [EMIM][FAP]/P(VDF-HFP) ionogel electrolytes containing LiPF_6_ were reported by Pandey and Hashmi (2013). They found that the specific capacitance of EDLCs with carbon nanotube (CNT) electrodes could be enhanced evidently by adding Li-salt into ionogel electrolytes. Using P(VDF-HFP) as a polymer skeleton, Shen et al. prepared [EMIM][NTF_2_]/P(VDF-HFP)/SiO_2_ ionogel electrolytes with high ion conductivity (Shen et al., 2015). The assembled [EMIM][NTF_2_]/P(VDF-HFP)/SiO_2_-based asymmetric microsupercapacitors with graphene quantum dots and MnO_2_ electrodes exhibited superior rate capability at 2,000 V s^−1^ and ultrafast frequency response (τ_0_ = 206.9 μs) owing to the enhanced ion conductivity of the fabricated ionogel electrolytes and fast ion diffusion. Moreover, some other polymers were also studied. For example, Kadokowa et al. synthesized a gel complexed with an IL (1-allyl-3-methyl-3-methylimidazole amide) and chitin/cellulose (Takegawa et al., 2010). Recently, an ionogel electrolyte based on graft copolymer (PEGBEM-g-PAEMA) was reported by Kang et al. Up to 200% [EMIM][BF_4_] content could be achieved in the fabricated electrolyte, and the assembled flexible supercapacitors showed better performance than other polymer/[EMIM][BF_4_]-based capacitors (Kang et al., 2020).

In addition, P(VDF-HFP)-based ionogel electrolytes also show a wide range of applications in quasi-solid LICs. Jiao et al. fabricated P(VDF-HFP) (Kang et al., 2020)/LiTFSI/EMIMBF_4_ ionogel electrolytes with a thickness of ~50 μm via a solution-casting method, which could widen the working potential of the assembled T-Nb_2_O_5_/rGO//AC LICs to 4 V, and thus, the energy density of 70 W h kg^−1^ at 1 kW kg^−1^ could be achieved at 60 °C (Jiao et al., 2018). The similar ionogel electrolytes but with different amounts of Li-salt and ILs were reported by Zhang J. H. et al. (2019). A quasi-solid LIC was obtained using P(VDF-HFP)/LiTFSI/EMIMBF_4_ as electrolytes and (Nb_2_O_5_@C)/rGO and AC as electrodes, showing a specific capacitance of 46.8 F g^−1^ at 1 A g^−1^ and a maximum energy density of 101 W h kg^−1^ at 60 °C (Figure 10B). From the shape of CV curves, a combination mechanism of fast adsorption on cathode and the sluggish Faradaic reaction in anode could be concluded. In addition, the effect of temperature on the charge transfer resistance (*R*_ct_) of the obtained device was also investigated, which showed a significant decrease in *R*_ct_ as the temperature rose.

Ionogel electrolytes could be used as flexible electrolytes and they determined the performance of quasi-solid flexible supercapacitors such as operating voltage window, stability, and rate capability. Sanjeev Kumar et al. designed flexible EDLCs with graphene electrodes using P(VDF-HFP)/EMIMBF_4_ ionogel electrolytes, delivering high capacitance of 242 F g^−1^ at 5 mV s^−1^ and long cycle life (Ujjain et al., 2015). In addition, a flexible sandwich EDLCs based on PS–PEO–PS/[EMIM][NTf_2_]/PTFE ionogel electrolytes and carbon nanotube electrodes was constructed by Kang et al. (2016). Such ionogel electrolytes with PS–PEO–PS host could self-assemble to form a nanostructure due to the hydrophobicity of PS segments and hydrophily of PEO segments, which would enhance the ionic conductivity. More recently, Pan et al. represented a fiber supercapacitor utilizing P(VDF-HFP)/EMIMTFSI ionogel electrolytes (Pan et al., 2019). The as-assembled solid fiber supercapacitor could be operated at 3.5 V, and thus, an ultrahigh energy density of 61.2 mW h cm^−3^ could be achieved.

Although the safety issues are suppressed using gel electrolytes instead of liquid electrolytes, the thermodynamic properties and mechanical stability are far from ideal. One promising approach is to construct hybrid IL gel electrolytes with inorganic substances. Moreover, the performance of hybrid IL gel electrolytes can still be further enhanced by selecting modified ceramics and oxide-based materials as a support. For instance, hybrid IL gel electrolytes composed of cage-structured polyhedral oligomeric silsesquioxanes (POSS), amine-terminated polypropylene glycol, ILs, and LiTFSI were reported, indicating that POSS could enhance the mechanical stability while the polyetheramine offers epoxy network structures linked through Li^+^ dissociative polyether linkages (Na et al., 2017).

### Poly(Ionic Liquid)-Based Ionogel Electrolytes

**Figure 11:**
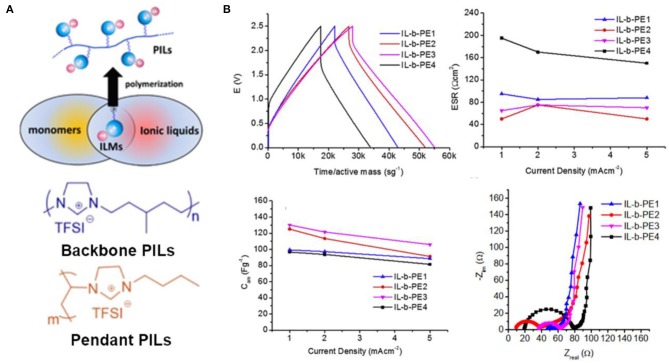
**(A)** Illustration of the structure of poly(ionic liquid)s (PILs) and molecular structures of backbone PIL and pendant PIL. **(B)** The electrochemical performance of solid-state supercapacitors assembled with the four electrolytes containing poly(diallyldimethylammonium) bis(trifluoromethanesulfonyl)imide (PILTFSI) and different ILs (Pyr_14_TFSI, Pyr_14_FSI, Pyr_14_DCA, and HEMimTFSI). Reproduced from Tiruye et al. (2016), Elsevier.

The history of PILs can be traced back to 1970s when the corresponding ionic organic salts undergone free radical polymerization. PILs are composed of a polymer backbone and anionic and cationic groups in monomer repeating units, which combine the properties of polymers and ILs, as shown in Figure 11A. In common synthesis processes, PILs are embodied in backbone PILs or pendant PILs depending on where the ionic species bind to the polymer chain, as illustrated in the following Figure 11A (Kuray et al., 2019). No matter what type of PILs, the transport of ions is achieved by the migration of the ionic species that can move freely. PILs can be utilized as the polymer hosts of ionogel electrolytes for supercapacitors. Compared to conventional polymers, PIL-based ionogel electrolytes can be regarded as superior solid electrolytes for solid supercapacitors, which possess advantages of high miscibility between PILs and ILs and high interactions of ions and PILs.

In the past years, the reviews on PILs in terms of their synthesis, chemical structure, physical properties, and applications were available (Lu et al., 2009; Mecerreyes, 2011; Yuan et al., 2013). Nevertheless, there are only limited reports related on PIL/IL electrolytes for supercapacitors. For instance, PIL/IL ionogel electrolytes containing poly(diallyldimethylammonium) bis(trifluoromethanesulfonyl)imide (pDADMATFSI) and their corresponding ILs were synthesized and applied to solid-state supercapacitors (Tiruye et al., 2015). The experimental results indicated that the voltage potential could achieve 3.5 V due to the high electrochemical stability of this PIL electrolyte. Further work fabricated four IL-based polymer electrolytes blending a PIL, poly(diallyldimethylammonium) bis(trifluoromethanesulfonyl)imide (PILTFSI), with four different ILs (Tiruye et al., 2016). The results of this work revealed that ionic conductivity was significantly aggrandized when the ILs contain small size anions (FSI^−^ and DCA^−^) than bigger anions (TFSI^−^), as shown in Figure 11B. In this way, the specific capacitance of assembled supercapacitors could be improved by increasing the ionic conductivity. In addition, recent works about [PIL-M-(Br)] and [PIL-M-(TFSI)] solid electrolytes composed of PIL and ILs were reported by Wang et al. (2018). Two solid electrolytes exhibited maximum energy density of 107 and 59.9 W h kg^−1^ when they were used for symmetric supercapacitors, respectively.

Previous studies have shown that the addition of ILs during the preparation of PIL electrolytes could increase the ionic conductivity of electrolytes. However, Yanga et al. prepared an anion-conducting poly(VAC-co-EVImBr) thin-film electrolyte without additional ILs, and a 4.2 V flexible all-solid-state C/C supercapacitor was assembled (Yang et al., 2017). The aim of this work was to demonstrate the feasibility of using inorganic anion-dominated conducting polymers as solid polymer electrolytes. More importantly, by comparing two types of solid polymer electrolytes, the cationic and non-ionic copolymer poly(VAC-co-EVImBr) is more rational than the cationic polymer PEVImBr. Furthermore, ILs cannot only be used as electrolytes directly but also can modify electrode materials. Ponkratov et al. proposed two families of PILs via different methods: one synthesized by free radical copolymerization was applied as electrolyte due to its high ionic conductivity; the other obtained by polymer modification and ion exchange was used as the components of the thin electrode films owing to their good ability to form coatings (Ponkratov et al., 2018). Based on this, all-polymer solid-state flexible supercapacitors with thickness of 480–500 μm were assembled using PILs as electrolytes. Although the performances of the assembled flexible supercapacitors were unsatisfactory, the proof-of-concept demonstration of novel flexible configuration was confirmed by this work for the first time.

## Conclusion and Future Perspective

Seeking for or formulating electrolytes with widened electrochemical window is a vital research orientation for constructing high-performance supercapacitors. According to the published research reports, IL electrolytes show enormous potentials in this field. Pure ILs system is significantly different from traditional aqueous and organic electrolytes. The energy storage mechanisms of supercapacitors with ILs need to be reconsidered. Conventional IL electrolytes enable the widened voltage windows of supercapacitors but may not lead to enhanced capacitance due to the difficulty in separating ions. Selecting and manipulating IL electrolytes reasonably according to diverse operating conditions and requirements are necessary on account of different characteristics of various IL electrolytes. Furthermore, the electrode structures affect the arrangement of ILs at the interface. A layered structure is formed at the planar electrodes, while a monolayer structure is formed on porous electrodes with proper pore size and a multilayer structure is formed on porous electrodes with wider pores. Nanoporous electrodes matching well with ion dimension of ILs exhibit the best performance under certain conditions. More recently, the properties and special interfacial nanostructures of self-assembly SAILs were demonstrated, which would be favorable for EDL structures and charge storage of EDLCs. High temperatures and wide voltage windows are advantageous for the application of supercapacitors with SAILs. Unlike conventional ILs, the unusual interfacial structure of SAILs results in enhanced capacitance. Mixing ILs with organic electrolytes can improve conductivity and durability in extreme environments. Obviously, this approach sacrifices the voltage window of IL electrolytes, and thus, the energy density of supercapacitors will be limited. Eutectic IL mixtures electrolytes may be favorable for advanced supercapacitors over a wider temperature range. Although great progress has been made in ILs/organic solvents and eutectic IL mixtures electrolytes, pure IL electrolytes sometimes exhibit better performance. Therefore, it is highly desirable to exploit universal strategies to tune the properties of the existing IL systems without the sacrifice of the voltage window and explore novel versatility of IL electrolytes for supercapacitors.

Besides liquid-state electrolytes, ionogel electrolytes may be a dominating tendency for future research due to the requirements of safe and high-voltage electrolytes for prosperous solid and flexible supercapacitors. Ionogel electrolytes based on conventional polymers and PILs have been extensively explored. Various strategies including the preparation of PILs/ILs and anion-dominated conducting polymers have been proven to construct advanced solid supercapacitors. More efforts need to be devoted to regulating the nature of IL electrolytes and the interface compatibility between electrolytes and electrodes. Ionogel electrolytes offer better interface compatibility and higher ionic conductivity but poor mechanical stability in comparison with solid polymer electrolytes. Inorganic substances may improve their mechanical stability.

**Figure 12:**
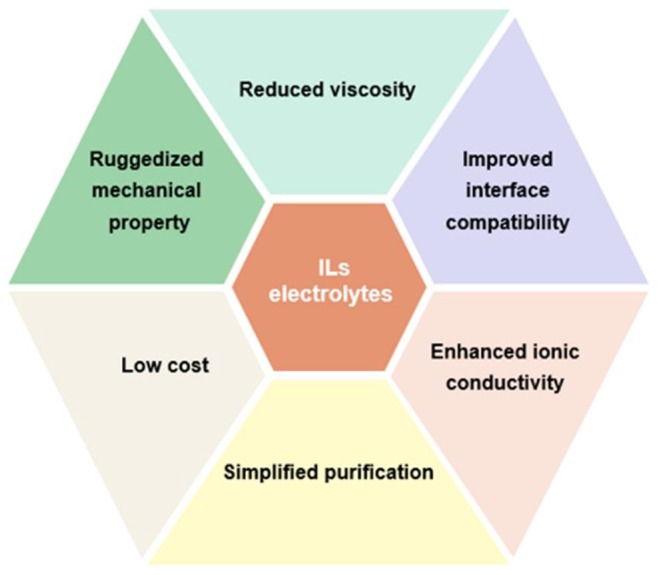
Challenges and future perspectives of ionic liquids (ILs) electrolytes for advanced supercapacitors.

Although previous research has proposed various strategies to settle these shortcomings of IL electrolytes, there still remains some problems needed to be further addressed such as high cost and high viscosity, as shown in Figure 12. Additionally, ILs exhibit poorer power performance than that of organic electrolytes. It is controversial whether ILs can replace traditional organic electrolyte of commercial supercapacitors. Despite these disadvantages, ILs as high-voltage electrolytes for supercapacitors promote research surge in constructing high-energy-density supercapacitors. The fundamental investigations of IL electrolytes is still essential to comprehend the basic characteristics of ILs, which is favorable for the development of safe and high-voltage electrolytes, thereby enhancing the performance of supercapacitors. Therefore, devoting more efforts to IL electrolytes is still needed to achieve advanced supercapacitors for energy storage.

## Author Contributions

All authors drafted the manuscript and approved it for application.

## Conflict of Interest

HZ was employed by company *Hebei Institute of Process Innovation Co. Ltd*. The remaining authors declare that the research was conducted in the absence of any commercial or financial relationships that could be construed as a potential conflict of interest.
